# Migration for Better Jobs or Better Living: Shifts in China

**DOI:** 10.3390/ijerph192114576

**Published:** 2022-11-06

**Authors:** Shuo Yang, Tianheng Shu, Taofang Yu

**Affiliations:** School of Architecture, Tsinghua University, Beijing 100084, China

**Keywords:** population migration, motivation, job, living, China

## Abstract

Traditionally, studies of mobility follow two main strands: migration driven by better jobs and by better living. However, the interactions and shifts between them are rarely addressed. In the largest-scale domestic migration, millions of Chinese movers have experienced changes in migration motivations in the past ten years. Starting with migration patterns, we attempt to explore the interaction and changes in migration motivations in a dynamic way and relate them to the evolution of socio-economic contexts. Based on the latest two population censuses (2010 and 2020) in mainland China, we built an empirical model attributing migration motivations to job and living conditions, and then estimated the model by ordinary least squares (OLS) and quadratic assignment procedure (QAP) methods. The results reveal that employment is the primary and fundamental factor, though its impact is weakening. Good living is becoming significantly influential in migration willingness, and there is an interactive effect between the factors of job and living. Furthermore, we offer an explanation of the motivation evolution as being migrants’ response to socio-economic status to maximize their utility. This study contributes to the migration literature from a longitudinal lens, and appeals to a continuous focus on migration evolution in the scientific research on population geography.

## 1. Introduction

Taking advantage of convenient transportation facilities and information channels, the increasing scale of population migration is shaping the world’s urbanization processes. International migration reached 281 million in 2020 [1], accounting for 3.7% of the world’s population. The seventh National Census of China shows that the total floating population reached 376 million in 2020, a significant increase of 155 million compared with the sixth National Census in 2010 (221 million), or nearly 70% growth [2]. Large-scale population migration is one of the most profoundly changing and socio-economically influential phenomena in China since the reform and opening up in 1978. Currently, China’s urbanization rate has climbed to 63.89%, and it has entered the middle and late stage of the transformation and enhancement of high-quality urbanization development [3]. In this process, population migration has become the predominant factor in the growth of urban dwellers, and is undergoing unprecedented changes [4]. Along with the tremendous population flow, destination sites of migration see massive crowdedness and exorbitant house prices as well as productive labor; meanwhile, the origin sites suffer from aging and economic stagnation due to population loss [5,6]. Therefore, it is necessary to analyze the patterns and motivations of population migration to help monitor and manage the floating population so as to guide migration in an orderly manner.

So far, researchers have identified various influential factors on migration motivations. The factors mainly belong to three categories: individual characteristics, economic factors (or jobs), and living-related factors. There has been considerable research on the impact of employment on migration [7,8,9], and the factors of living have been studied in recent decades [10,11]. Living or living condition is a comprehensive concept regarding the provision of social services, environment contamination, diversity, etc. [10,12,13]. Intuitively, people tend to live in places with good living conditions because this is likely to provide a better sense of happiness. Although some scholars hope to integrate the two factors of employment and living (see Section 2), there are still few studies exploring the interaction between the two motivations. Moreover, most preexisting literature tested these factors statically based on cross-section data, but in fact, their influences may change over time, especially against an evolving socio-economic background. Economic depression has lowered people’s expectation of more income by changing jobs, and the COVID-19 pandemic may draw attention to sufficient medical resources. Will economic factors dominate migration decisions as powerfully as before? How and to what extent will migrants value their living conditions? Such developments warrant migration studies with a dynamic aspect.

To fill the research gap, this paper analyzes the changes in migration patterns and motivations in China from 2010 to 2020, and reveals how socio-economic evolution impacts these changes. This study makes contributions to the scarcity of studies in longitudinal motivation shifts and associated influential socio-economic factors, considered as a reference for future research on migration shifts amid changing social circumstances.

The remainder of this paper is organized as follows. The second section reviews related work. The third section introduces the model and data. The fourth section describes the evolution of migration patterns and reports the model results, followed by further discussions in the fifth section. The last section summarizes the conclusions, offers several policy implications, and states the limitations.

## 2. Literature Review

To date, migration theory has always been evolving with socio-economic development, and scholars continue to update their focus on migration motivations. De Hass pointed out three typical stages in migration after World War II [14]. In the 1950s and 1960s, under the neo-classical economic theory, development economists regard migration as labor transfer, influenced by supply and demand among origins and destinations [7]; therefore, the gap in labor prices, or the wage gap, was deemed the main factor [8]. This is followed by the growth theory, which focuses on the productivity change after the laborers move to their destination and find a new job [9]. Opposite to this optimistic view, the neo-Marxist development theories in the 1960s blamed migration on human capital exploitation in poor origin areas for the benefit of developed destinations [14,15], such as the brain drain [16]. Characteristics of destination countries received much attention from scholars at that time [16]. Since the 1990s, migration studies have entered a pluralist stage [14,15], with two distinguished approaches: New Economics of Labor Migration (NELM) [7,17] and the Sustainable Livelihood Approach (SLA) [18,19]. NELM emphasizes the influence of migrants’ remittance [17,20], and nowadays, some scholars consider these monetary motivations too simplified, and put forward that people, especially well-educated elites, move for a non-monetary fulfillment from career success [21,22]. However, SLA studies claim that access to resources (education, health care, or well-being) and avoiding vulnerability [23,24] are as important as economic factors [15,18,25]. As Maslow noted, besides physiological and safety needs, people have social, esteem, and self-actualization needs [26]. For example, unlike economic motivations, many rural teenagers move to cities out of curiosity, seeking freedom and diversity [27]. Adults would move for better care for their family [28]. For example, people tend to move to be with their spouses [13], or to let their children attend better schools [29]. In summary, better consumption and service quality in cities meet movers’ high-level needs above survival, and thus make cities attractive destinations. Recently, there emerged an attempt to integrate NELM and ASL [14,15], indicating a trend that economic and well-being motivations should both be valued.

Along with the theory evolution, researchers have empirically identified various motivations, mainly falling into three categories: individual factors, career pursuit (or good jobs), and well-being [11,30,31]. Among individual-level studies, age, gender, marriage, children, household, etc., have been carefully tested by microeconomic data [11,32]. As for socio-economic context, employment status and income have been recognized as fundamental variables in motivation research [31,33]. However, living-related factors such as public services, safety, and climate, though quite valuable for residence [13,34], have not been explored adequately. Aslany et al. performed a systematic review of migration aspirations, based on 49 representative publications sifted from 438 publications regarding migration, recording all the factors of every survey involved [11]. Among them, 35 publications involve employment, 19 involve living conditions, and only 13 involve them both. In recent years, with climate change bringing a rise in migration incidence and mortality rate, migrants and scholars are growing more concerned with suitable living environments [23,35]. Thus, more efforts are required in the correlation of migration and living conditions, as are further empirical tests on economic and living-related factors together.

Driven by factors varying among regions, migrations have presented different spatial patterns, such as migration from rural to urban areas, urban to rural areas, inland cities to coastal cities, etc. [36,37]. These migration patterns are usually spatially uneven and may change over time, e.g., the emergence of counter-urbanization in coastal areas [38]. In the Chinese context, many studies have also analyzed migration patterns and willingness to migrate using cross-sectional data from the National Health and Wellness Commission’s Mobility Monitoring Survey [6,39]. However, most cross-sectional data employed are static, and longitudinal comparisons remain scarce. Moreover, due to data availability, there are limited systematic descriptions of migration patterns and their characteristics in nationwide views.

To summarize, the preexisting research mostly sheds light on the identification of multiple motivations of migration, while the influence of living-related factors and the interactions between economic and living-related motivations remain an open question, and the spatial evolution of population migration patterns in China is yet to be adequately addressed. Furthermore, the temporal evolution of motivations and related factors have been less discussed. Indeed, the overall migration preferences may change in different periods due to the transformation of people’s recognitions, living pursuits, spiritual needs, etc., as well as demographic adjustments and socio-economic context [33,40,41]. Typically, unlike their elders, many new generations (born after the 1980s) are reluctant to pursue over-loaded work though with a high salary, instead drawn to jobs in line with their interests [27]. In recent years, the economic depression has spread among countries, and China, even with strong economic performance, is facing a significant drop in GDP growth from 10.6% (2010) to 2.2% (2020). Then, would the pessimism expectation of economic and salary growth decrease movers’ economic motivation? Additionally, the spread of COVID-19 shows the disadvantage of high-density metropolises, conflicting with traditional preferences for urban areas [42,43]. These evolutions of social conditions are appealing for a dynamic aspect of migration motivation changes, which this paper investigates.

## 3. Method and Data

### 3.1. Model Specification

The core objective of this paper is to explore the motivation changes for population migration in China. We use the regression model to identify the influence of potential factors on migration. Building on the existing literature, excluding personal characteristics, there are two main categories related to economic and living conditions, which contain factors such as employment opportunities, environment, education, health care, etc. [4,6,40,41]. Considering data availability and indictor representativeness, this paper focuses on two core factors (determinants) that are widely recognized by the academic community, i.e., job opportunities and public service resources, also highlighted in the literature review section. Many people move to find a new job with higher income or greater achievement, and this is closely associated with local economic conditions [44]. As for the second group of people seeking better living conditions, although they can obtain non-local goods by delivery, they have to rely on local facilities for health care and children education services. Academically, numerous studies have proved the value of these facilities in promoting living conditions [34]. For example, Zhou et al. [45] demonstrated that the better the public service facilities are, the happier the people are. In other words, the level of public services such as health care and children’s education directly reflect the conditions of living in a region. As such, we consider incorporating the two factors of job opportunities and public services into our analysis model. To be specific, we use the differences in job opportunities, health care, and education levels across locations as the proxy of motivations for migrators, among which health care and education levels represent the living conditions. In addition, the willingness to migrate decreases as the migration distance increases. We add the population size factor and the terrain factor (e.g., mountainous areas) to control for the main confounding factor. Based on the above discussion, we construct a linear regression model as follows.

(1) $$ln\left(Flow_{i,j}\right)=\alpha \cdot \Delta Job_{i,j}+\beta_{1}\cdot \Delta Heal_{i,j}+\beta_{2}\cdot \Delta Edu_{i,j}+\gamma_{1}\cdot \Delta Job_{i,j}\cdot \Delta Heal_{i,j}+\;\gamma_{2}\cdot \Delta Job_{i,j}\cdot \Delta Edu_{i,j}+\delta \cdot Dist_{i,j}+\theta \cdot ln\left(Pop_{i}\right)+\eta \cdot Terr_{j}+\varepsilon$$

where $Flow_{i,j}$, as the explained variable, is the number of migrators who move from origin *i* to destination *j*. $\Delta Job_{i,j}$ is the gap in jobs (salary and opportunities) between *j* and *i*, which is approximated by the difference in gross domestic product (GDP) per capita [46]. $\Delta Heal_{i,j}$ and $\Delta Edu_{i,j}$ are the gaps in health care and education levels, which are measured by the differences in the number of hospital beds per thousand residents and the number of registered students in elementary schools per 1 million residents, respectively. $Dist_{i,j}$ is the geographical distance (10^3^ km) from origin *i* to destination *j*. $Pop_{i}$ is the population amount in origin *i*. $Terr_{j}$ is a dummy variable indicating whether the destination terrain is suitable for settlement. At last, to test whether job opportunities and living conditions have an interactive effect on migration, two interaction terms between job opportunities and living conditions, $\Delta Job_{i,j}\cdot \Delta Heal_{i,j}$ and $\Delta Job_{i,j}\cdot \Delta Edu_{i,j}$, are added to the model. ε remains a random error term.

In model (1), education and health care are treated as separate variables. Previous studies demonstrate that living-related factors exhibit different levels of importance in people’s migration decisions [6,11,21]. However, in this study, we aim to build the relation between socio-economic evolution and changes in migrants’ attitude towards living conditions. Therefore, we need a comprehensive proxy of living conditions and thus use principal component analysis (PCA) to integrate the information of $\Delta Heal_{i,j}$ and $\Delta Edu_{i,j}$, and then we obtain one composite variable, $\Delta Living_{i,j}$. PCA is a method of dimensionality reduction, which enables creating a single metric from a suite of indicators without important information loss. Hence, model (1) can be modified as follows:

(2) $$ln\left(Flow_{i,j}\right)=\alpha \cdot \Delta Job_{i,j}+\beta \cdot \Delta Living_{i,j}+\gamma \cdot \Delta Job_{i,j}\cdot \Delta Living_{i,j}+\;\delta \cdot Dist_{i,j}+\theta \cdot ln\left(Pop_{i}\right)+\eta \cdot Terr_{j}+\varepsilon$$

where the variables hold the same meaning as model (1). For models (1) and (2), we adopt the OLS method to estimate the coefficients of independent variables.

### 3.2. Research Area and Data

**Figure 1:**
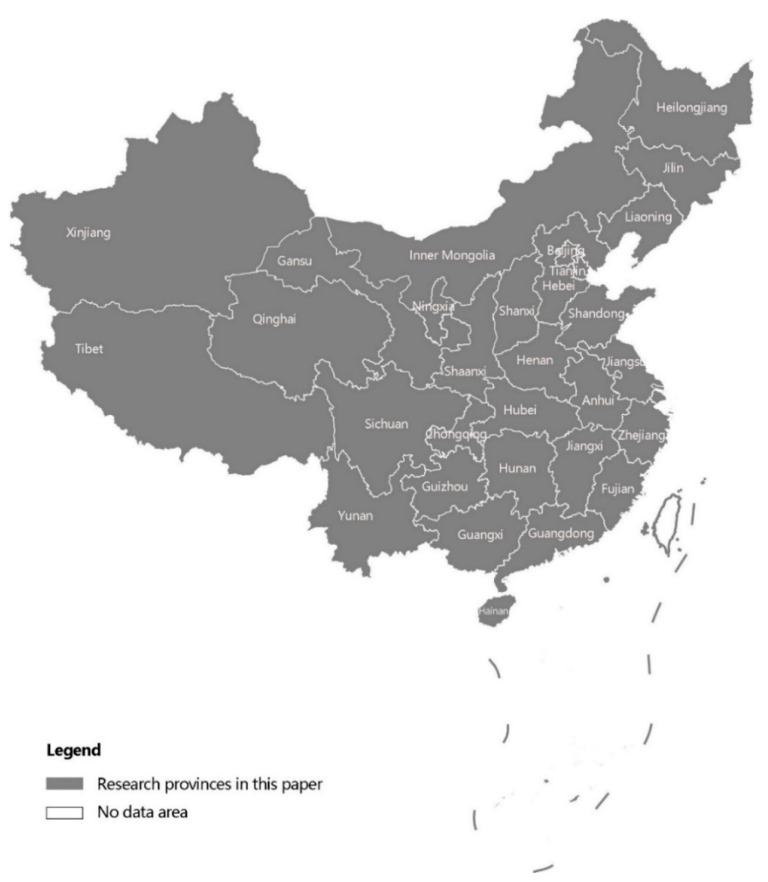
Study area.

In this study, we focus on the migration changes among urban and rural areas in 31 provinces of mainland China during the period of 2010–2020. The research area is shown in Figure 1.

Owing to the differences in data statistical standards among yearbooks, it is impossible to follow a consistent analysis unit from the existing data sources. Alternatively, we regard Qu units (third-level administrative divisions in China) and Jiedao units (fourth-level administrative divisions) as urban areas, and consider county units (third-level administrative regions) and town or village units (fourth-level administrative divisions) as rural areas. We believe that this division is reasonable in that it reflects the gap between rural and urban areas in the Chinese economic and facility establishment context. Thus, we can quantify four migration patterns of Chinese population migration, namely rural-to-urban, rural-to-rural, urban-to-urban, and urban-to-rural flows. Following this rule, we can map the spatial patterns of migration in China in 2010 and 2020.

We extracted migration data $Flow_{i,j}$ and population data $Pop_{i}$ among urban and rural areas in 31 provinces from *China Population Census Yearbooks* for the years 2010 and 2020. Data on GDP, hospitals, and elementary school students came from *China Health Statistics Yearbooks* and *China County Statistical Yearbooks*. For several missing data, we collected them from relevant websites (e.g., the website of Statistics Bureau of Xinjiang Uygur Autonomous Region, http://tjj.xinjiang.gov.cn/ (accessed on 15 September 2022)). Finally, with the three kinds of data divided by population, we obtained the data of GDP per capita (10,000 yuan), the number of medical beds per 1000 people, and the amount of elementary students per 1 million people as proxies of local jobs $Job_{i}$, health care levels $Heal_{i}$, and education levels $Edu_{i}$. Then, using the PCA method, we calculated the covariance matrix of $Heal_{i}$ and $Edu_{i}$ (2010 and 2020 together) and its eigenvectors (0.808, 0.589) and then derive $Living_{i}$ by combining $Heal_{i}$ and $Edu_{i}$ with the weights of 0.808 and 0.589. Next, the gaps in jobs between destinations *j* and origins *i* can be derived by $\Delta Job_{i,j}=Job_{j}-Job_{i}$, as our explanatory variable. The same applies for the gaps in living-related variables. The migration distance $Dist_{i,j}$ is estimated by the length from origin *i* to destination *j*. For provinces with the most areas of steep mountains or deserts (e.g., Tibet), we set $Terr_{j}$ as 1, otherwise 0. More details are listed in Table A1, Appendix B. The descriptive statistics of all variables are presented in Table 1.

Besides quantitative data, the *China Population Census Yearbooks* for the years 2010 and 2020 provides a summary of the reasons for migration derived from questionnaire surveys. The summaries in two years cover 85,876,337 and 124,837,153 migrants, respectively, with far larger magnitudes than sample surveys in previous research [6,39,47]. The reasons can be divided into three categories. The first is career, which encompasses job and skills training. The second is living conditions, which includes care for family members and obtaining a *hukou* (see explanation of *hukou* in Appendix A). Lastly, the third includes other factors, covering marriage, demolition, and other minor reasons. In Section 5.2, we use these data to further confirm our deductions from empirical findings.

## 4. Results

### 4.1. Spatial Characteristics of Migrations in China

**Figure 2:**
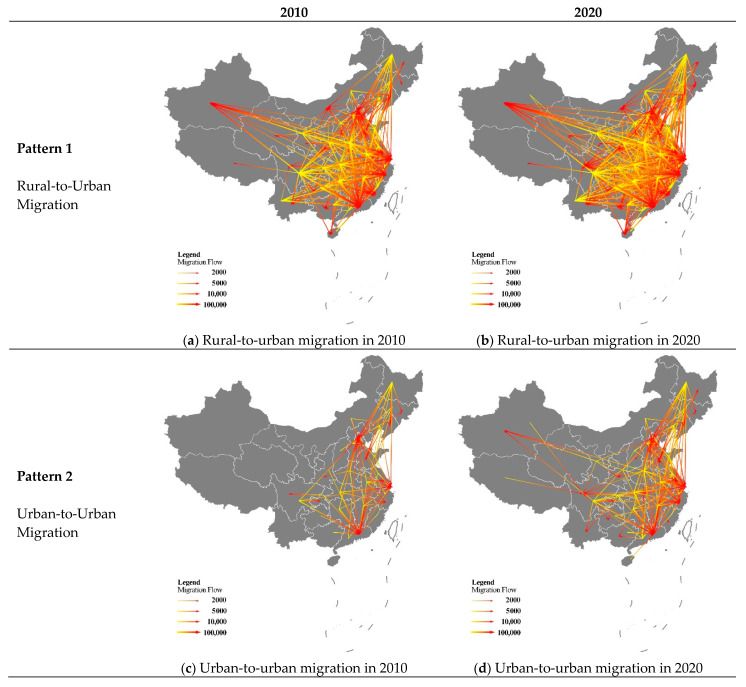

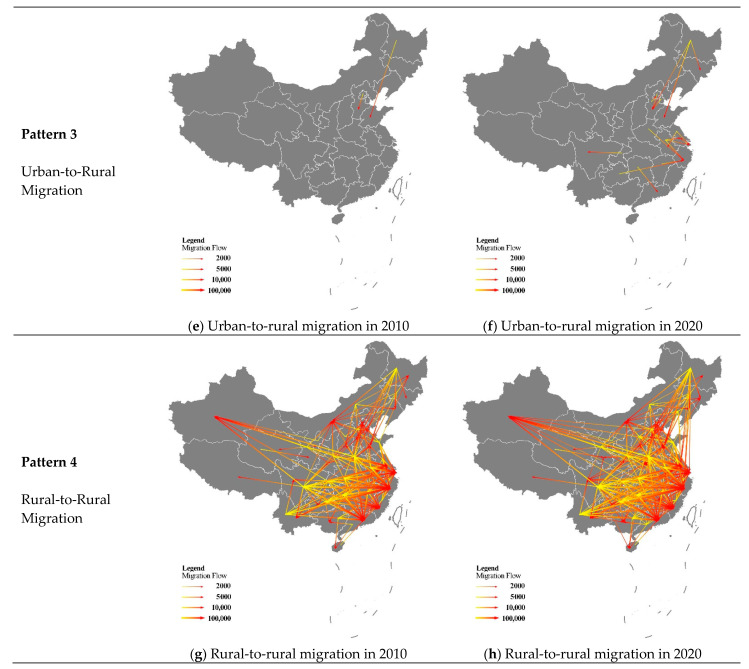
Four migration patterns in 2010 and 2020.

From 2010 to 2020, Chinese migration rose significantly in quantity and vastly spread. The amount of migration jumped by 85% (from 24 million to 45 million). In terms of spatial movements, rural-to-urban migration remained dominant among the four kinds of migration during the 10-year period. Figure 2a,b depict inter-provincial urbanization flows in the two censuses and show three remarkable points. First, Beijing, Shanghai, Zhejiang, Fujian, and Guangdong had the most attraction to movers, while people in Hunan, Henan, Heilongjiang, Jilin, and Sichuan had the largest tendency to move out over the 10-year period. Second, several new hotspots of immigration and emigration appeared in 2020. Guangxi and Hainan have been in a stage of people gathering, while Shaanxi, Shanxi, and Gansu began losing large amounts of population. Third, as a summary, the centroid of migration activities has been moving westwards, and the central provinces have experienced the most dramatic urbanization processes in mainland China.

The second large-scale migration is the horizontal movement between cities (Figure 2c,d). Similar to rural-to-urban migrations, from 2010 to 2020, urban migration activities were moving westward. On the one hand, following the migration feature of the northeastern region in 2010, Henan, Hunan, and Hubei joined the group of large-scale population outflow areas; on the other hand, Hubei, Sichuan, and Xinjiang became popular migration destinations. In terms of spatial location, the centers of population outflows and inflows happened to be the surroundings of the Beijing–Guangdong railway line, one of the busiest railroad trunk lines in China.

Next came the rise in counter-urbanization flows from urban to rural areas (Figure 2e,f). The year 2010 saw few large-scale urban-to-rural migrations, with only migrations from the colder cities of Heilongjiang to rural areas in Shandong and from Beijing to the neighboring Hebei province. The year 2020 saw two types of migrations. The first is migration from Beijing and Tianjin to the neighboring Hebei Province as a result of population deconcentration policies in megacities. The other category is long-distance migration from cities in Heilongjiang, Henan, Guizhou, and Hunan to towns and villages in Beijing, Shanghai, and Guangdong. Despite the move from cities to rural areas, these areas have the advantage of geographical proximity to metropolises such as Beijing, which provide better job opportunities compared to some developing and small cities.

The spatial distribution of migration flows among rural areas did not change substantially (Figure 2g,h). This may be accounted for by the low development progress compared to urban regions. Many migrators born before the 1980s in villages have little interest in city life but aim directly at the manufacturing factories far away from cities to earn money for their families. Consequently, they repeat their familiar routes year by year.

### 4.2. Choices of Destinations

**Figure 3:**
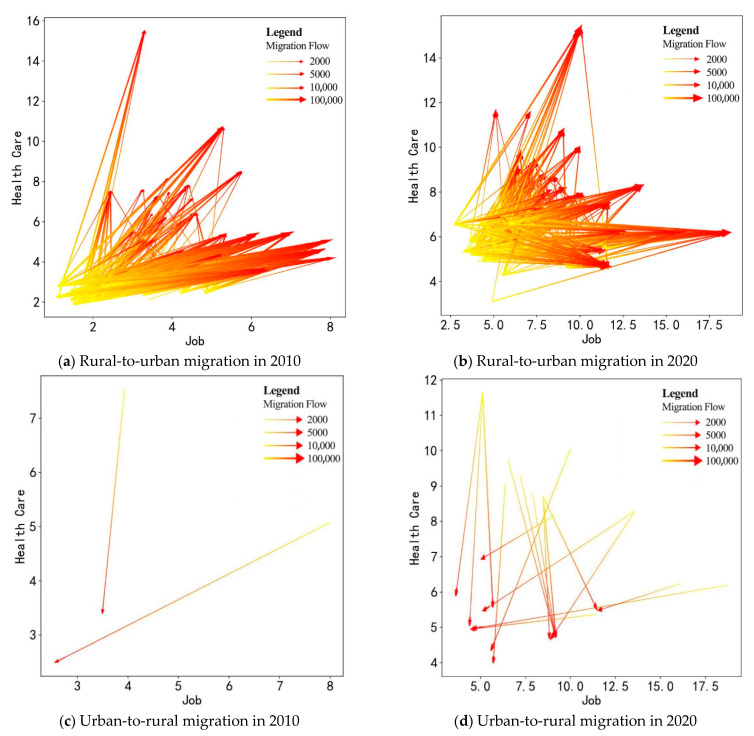
The economic and health care conditions of destinations in 2010 and 2010.

Following the above understanding of the spatial distribution of migration flows, we conduct an in-depth analysis on destination choices, considering their economic and social conditions (here, we use GDP per capita to measure the level of economic development, and health care resources per capita to represent social services capacity (thus living quality)). We selected two typical migration patterns for analysis, namely the urbanization process of rural-to-urban migration and the counter-urbanization process of urban-to-rural migration. The lines in Figure 3 indicate the distribution of migration flows from origins (yellow) to destinations (red). As illustrated in Figure 3a,b, destination choices of rural-to-urban migrations changed significantly over 10 years. In 2010, most people migrated to places with higher GDP per capita and a slightly higher standard of living than their origin. In 2020, GDP per capita in many destinations is not that much higher than in origins, but the public service capacity is significantly better than in origins. In contrast, in the counter-urbanization migration of 2020, the level of economic development is considered fundamentally important (Figure 3c,d). Migrants may accept a decrease in public service levels, but the huge drop in economic conditions in destinations would be undesirable. Thus, we can preliminarily conclude that economic development (reflecting job opportunity) is a fundamental consideration in the choice of destination for migration, but perhaps migrants have been placing more emphasis on living quality than before.

### 4.3. Motivations of Migration in China

We adopted the OLS model specified in Section 3.1 to define the driving forces of migration in China. We ran the OLS model for each separate year to detect the dynamics of the driving forces. Before that, we calculated the variance inflation factor (VIF) to diagnose the multicollinearity among independent variables (see Table 2). The VIF values for all regressors are less than 10, indicating that the issue of multicollinearity in our model is negligible. We use robust standard error to mitigate the influence of potential heteroscedasticity and autocorrelation.

Using model (1), we estimate the contribution coefficients of each factor in the choice of migration destinations. We find that employment is an essential determinant of migration, significantly positive at the 0.01 level (columns 1–3 and columns 5–7 of Table 2), and its coefficients are much larger than those of living factors. However, we notice a significant decline in the contribution of job opportunities over the 10-year period, from 0.275 (column 3) to 0.179 (column 7), indicating a decrease in the demand for higher incomes among migrators when choosing migration destinations.

People are assigning more importance to living conditions in their migration choices. For health care resources, the coefficient in 2020 (0.024) is larger and more significant at 1% than in 2010 (0.017) at the 5% level (columns 2 and 6). After adding the education variables, the coefficients of health care and education in 2010 show opposite signs, indicating that migrants do not have a clear preference for living conditions (column 3). However, the results in 2020 do not show significant conflict (column 7). Thus, we can deduce that migrants in China assign more importance to health care over the 10-year period.

Third, columns (2), (3), (6), and (7) all report significantly negative coefficients of the interaction term, suggesting that job opportunities and living conditions (mirrored by health and education level) have an interactive effect on the utility of migrants. In other words, prominent economic conditions (job opportunities) at a destination can reduce the contribution of good living conditions to the willingness of migration; it holds the same implications for superior living conditions. In addition, we notice that migration distance has a significantly negative impact on migration willingness (see columns 1–3 and 5–7 in Table 2), which is in line with the common sense that migration intentions decay as travel costs increase [48,49]. The absolute value of the coefficient of distance in 2020 is nearly 17% lower than that in 2010, which may suggest that the improvement of transportation facilities have weakened the distance limitation when people consider migration.

Next, we report the estimation results of model (2), as presented in Table 3. Herein, living conditions are a combination of health care and education conditions. We find that the combined living condition variable in 2020 is positively correlated with migration at a significant level of 1%, while that in 2010 is not significant, confirming the results of model (1). We also detect that there is a remarkable decline in the influence of jobs on movers’ willingness of migrating, by about 11% (=1 − exp (0.183)/exp (0.305)) from 2010 to 2020. Additionally, the coefficient of living changes from insignificant to significant (positive), which provides further evidence that migration in mainland China is increasingly driven by living conditions.

### 4.4. Robustness Check

We conducted robustness checks in two ways. First, we added additional control variables regarding natural disasters, as previous studies have shown that the incidence or potential threat of natural disasters in an area could lower migrants’ willingness to live there [35,50,51]. Therefore, in our basic OLS model, we added a dummy variable indicating whether there have been tremendous hazards in the past ten years at the destinations. If the province *j* suffered an earthquake or typhoon in 2000–2010, $Disaster_{j}=1$, otherwise 0 for the regression in 2010. Similarly, we set this variable in the regression of 2020 according to the hazard records in 2010–2020. The results are shown in Table 4. We can observe the declining influences of job and distance, the increasing emphasis of living conditions, and a significantly negative coefficient of the interaction term, coinciding with Table 3, indicating that the additional control variable $Disaster_{j}$ will not influence our main findings.

Second, as the explained and explanatory variables exist in migration networks, there could be potential bias for our estimation by multicollinearity generated from networks. The quadratic assignment procedure (QAP) can exclude these potential biases. Thus, we re-ran our model using the QAP method. The results are shown in Table 5. Excluding the observation dependence, the coefficients of job and distance remain significant at the 1% level and the coefficient of living conditions in 2020 is 10% significant, though with a drop in significance compared to Table 3. The interaction terms lose their significance, but their *p*-value is small and acceptable. Together with our robustness check above, we have confirmed our basic findings, and we discuss these results in the next section.

## 5. Discussion

### 5.1. Evolution of Migration Patterns

Of the four migration patterns, the two largest ones, rural-to-urban and urban-to-urban, were both moving westward, and urban-to-rural migration was on the rise and rural-to-rural migration changed slightly. These population flows are in accordance with industry transformation in China [52]. With China’s strategy of industrial upgrading and the central and western regions developing, traditional labor-demanding manufacturing industries are moving from the east to the central and western regions. Thus, a large number of urban labor forces are attracted to central and western cities, where rural young people shift to an urban population. The provincial capitals of Sichuan, Guangxi, and Hainan are all rapidly gathering population and industry. As a result, the distribution of the population is becoming more balanced among the east, central, and western regions. Meanwhile, within the region, counter-urbanization migration is balancing the population distribution between large cities and small counties. Small counties can also attract newcomers by their specialty industries and geographical proximity to larger cities. The town of Zhangjiang is a successful example of this.

### 5.2. Shift in Migration Motivations

**Figure 4:**
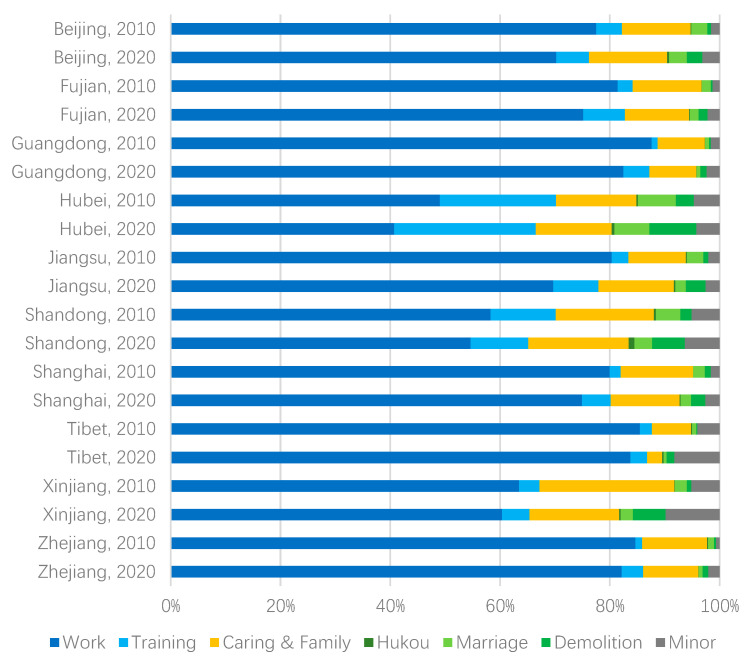
Migration motivation shares in the top ten migration provinces.

Our OLS estimates suggest that employment is the fundamental driver of population migration, but the quality of services such as health care and education are beginning to play an influential role in migration flows. This is in line with the basic theories of development economics and labor economics [15]. We further leverage the additional information (survey of migration motivations, see Section 3.2) of *China Population Census Yearbooks* [53,54] to shed light on the shift in migration motivations during 2010–2020. Figure 4 shows the share of motivations summarized from the census reports in the top ten migration provinces.

As shown in Figure 4, of the changes (2010–2020) in the three categories of migration motivations (i.e., career, living conditions and other factors, such as marriage, demolition, and other minor reasons), the most obvious is the decline in career factors. Of the ten provinces, Beijing had the largest decline of 6%. Although the share of skill training purposes increased in some cities, it tends to be a temporary stay of several years of apprenticeship and has limited impact on the overall trend of population mobility. The shares of living remain stable or increase in nine cities, which reflects the attractiveness of diversified services and facilities to the population, consistent with our findings from the regression model. Moreover, the shares of minor factors increased significantly, and the motivations for migration are more diversified than before. In other words, migrants are benefiting from migration in more aspects besides career and living conditions.

### 5.3. Relating the Shift in Migration Motivations to Socio-Economic Context

**Figure 5:**
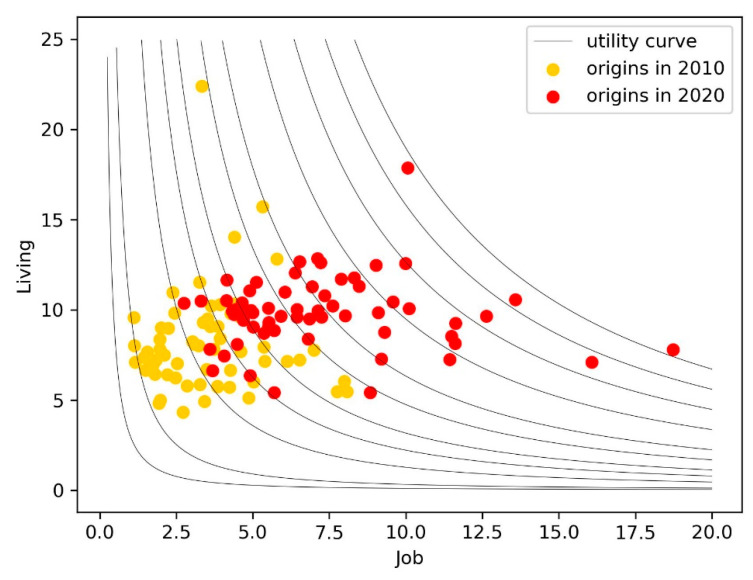
Utility curves and origin conditions of immigrants.

In the decade of 2010 to 2020, we argue that the changes in migration motivations are closely related to the changing context of the socio-economic environment. The most important factor can be attributed to the changes in consumers’ pursuits of maximum utility under rapid urban development. In the regression model, we have demonstrated that the coefficients of job opportunities and living conditions are positive, while the coefficients of the interaction term are negative. Hence, it can be deduced that the equal utility curve of migrants for job opportunities and living conditions goes from top left to bottom right, concave to the origin [55]. Figure 5 illustrates the equal utility curves and the changes in job opportunities and living conditions of these regions. In 2010, the GDP per capita was low everywhere, and the utility curve is almost perpendicular to the horizontal axis; thus, migrants pursued better jobs to maximize their utility. After a decade of economic development, GDP per capita almost doubled, and most scattering points shift rightward. At this point, the movement rightward and upward becomes the best choice to reach a curve of better utility, so migrants show more inclination to places with better living conditions as destinations to maximize their utility.

In addition to economic development, internet popularity has reduced the need to move for training and work. As to training, instructional videos and live streaming are growing at an unprecedented rate on various websites. For work, many tasks and meetings can also be conducted online—especially after the outbreak of COVID-19 in 2020, working from home has become normal. Therefore, moving for a new job does not seem as necessary as before. In contrast, health care and children’s education require more face-to-face interaction between clients and providers, and thus, the motivation to move for better living conditions has remained stable or even increased.

### 5.4. Impact of Migration Motivation Shift on Urbanization Processes

As the construction of transportation facilities in China are alleviating the constraints on long-distance movement, an increasing magnitude of migration is influencing urbanization processes. For a long time, some theories of economics such as development economics have stressed the huge attraction of urban areas to labor forces due to the urban–rural wage gap [8,15]. As a result, millions of farmers have flocked to urban industrial parks, and policy makers in large cities have been frustrated by failures to decongest overcrowded populations. Nowadays, many firms in industrial parks are experiencing “labor shortages”, even offering unprecedentedly high wages [56]. Many small cities are making efforts to improve health and recreation to attract newcomers. This indicates that the quality of living will become the focus of migration motivation studies. Moreover, governments can guide the reallocation of the population among large cities, small cities, and villages through the construction of service facilities to optimize spatial distributions.

## 6. Conclusions

Population migration is a key element in the realization of Chinese urbanization. Based on the data from China’s sixth and seventh population censuses, this study analyzed the evolution of spatial migration patterns and explored the migration motivations in mainland China as well as their dynamics from 2010 to 2020. Throughout the 10-year period, better employment has always been the determining factor, but its influence is weakening. On the contrary, living is becoming significantly influential when choosing migration destinations. This change is the result of migrants selecting new paths to gain maximum utility against the changes in the socio-economic environment. In addition, the construction of transportation facilities has relaxed the constraints on migration distance, leading to larger-scale movements and freer destination choices.

Our study has three main theoretical contributions to the existing knowledge. The first is that four migration patterns of the Chinese floating population are quantitatively portrayed and mapped, and the dynamics of the different migration patterns between 2010 and 2020 are explored. The second is that based on several identified migration motivations, this paper confirms that there is an interaction between different motivations; as an example, satisfactory living conditions at the migration destination can, to some extent, compensate for the inadequacy of job opportunities. Third, this paper reveals that the influences of jobs and living conditions on migration are not fixed, but change with the evolution of the socio-economic environment.

Our findings have two implications for policy makers. First, given the background of an aging population, attracting new residents, especially young laborers, has been essential for regional development. Since migrants are giving more consideration to living conditions, regional and city governments should invest more in a comfortable life, such as adequate public facilities and fresh air, rather than the choice of real estate as before. Second, the emergence of counter-urbanization flows offers a chance to relieve the over-crowded pressure on mega-cities such as Beijing. By improving living conditions in small cities nearby, regional governments could guide people to move outside of mega-cities and reach the goal of a balanced population distribution.

Several limitations of this study are acknowledged. Due to data availability, this study does not consider other factors which may confound our estimation, such as sports facilities and regional culture. Undoubtedly, the history of trades in certain regions will increase the population mobility, which brings bias to the estimation. Secondly, the traffic control against the COVID-19 epidemic (“do not go outside the province unless necessary”) also biases the regression estimates for our results from 2020. In addition to traffic constraints, the COVID-19 epidemic has raised greater concerns about health care resources among migrants, and whether this trend will affect migration flows in the long run will be a concern for future research.

## Figures and Tables

**Table 1:** Descriptive statistics for migration flows and related factors.

	2010			2020		
	N	Mean	Std. Dev.	N	Mean	Std. Dev.
$ln\left(Flow_{i,j}\right)$	3762	5.557	2.075	3779	6.277	1.950
$\Delta Job_{i,j}$	3762	0.007	2.434	3779	0.002	4.440
$\Delta Heal_{i,j}$	3762	0.011	3.648	3779	0.002	3.111
$\Delta Edu_{i,j}$	3762	−0.001	3.758	3779	0.001	2.775
$\Delta Living_{i,j}$	3762	0.008	4.038	3779	0.003	2.832
$Dist_{i,j}$	3762	1.471	0.790	3779	1.476	0.793
$ln\left(Pop_{i}\right)$	3762	16.455	1.057	3779	16.588	0.942
$Terr_{j}$	3762	0.224	0.417	3779	0.226	0.418

**Table 2:** The regression results of model (1).

Year	2010				2020			
	(1)	(2)	(3)	(4)	(5)	(6)	(7)	(8)
	$ln\left(Flow_{i,j}\right)$	$ln\left(Flow_{i,j}\right)$	$ln\left(Flow_{i,j}\right)$	VIF	$ln\left(Flow_{i,j}\right)$	$ln\left(Flow_{i,j}\right)$	$ln\left(Flow_{i,j}\right)$	VIF
$\Delta Job_{i,j}$	0.305 ***	0.296 ***	0.275 ***	1.78	0.183 ***	0.179 ***	0.179 ***	1.13
	(0.009)	(0.010)	(0.012)		(0.005)	(0.005)	(0.005)	
$\Delta Heal_{i,j}$		0.017 **	0.026 ***	1.58		0.024 ***	0.024 ***	1.08
		(0.007)	(0.007)			(0.007)	(0.007)	
$\Delta Edu_{i,j}$			−0.023 ***	1.68			−0.002	1.17
			(0.007)				(0.011)	
$\Delta Job_{i,j}*\Delta Heal_{i,j}$		−0.008 ***	−0.009 ***	1.01		−0.005 ***	−0.005 ***	1.04
		(0.003)	(0.003)			(0.002)	(0.002)	
$\Delta Job_{i,j}*\Delta Edu_{i,j}$			0.003	1.01			0.001	1.01
			(0.002)				(0.002)	
$Dist_{i,j}$	−0.963 ***	−0.962 ***	−0.964 ***	1.10	−0.799 ***	−0.795 ***	−0.796 ***	1.11
	(0.029)	(0.029)	(0.029)		(0.027)	(0.027)	(0.027)	
$ln\left(Pop_{i}\right)$	0.893 ***	0.880 ***	0.884 ***	1.14	0.946 ***	0.936 ***	0.937 ***	1.06
	(0.021)	(0.022)	(0.022)		(0.022)	(0.022)	(0.022)	
$Terr_{j}$	−0.484 ***	−0.504 ***	−0.470 ***	1.21	−0.608 ***	−0.619 ***	−0.616 ***	1.17
	(0.054)	(0.056)	(0.057)		(0.051)	(0.052)	(0.053)	
Constant	−7.612 ***	−7.364 ***	−7.424 ***		−8.092 ***	−7.923 ***	−7.918 ***	
	(0.356)	(0.370)	(0.371)		(0.382)	(0.383)	(0.392)	
Observations	3762	3762	3762		3779	3779	3779	
R-squared	0.587	0.589	0.590		0.580	0.582	0.582	

Robust standard errors are in parentheses. *** *p* < 0.01, ** *p* < 0.05, * *p* < 0.1.

**Table 3:** The regression results of model (2).

Year	2010		2020	
	(1)	(2)	(3)	(4)
	$ln\left(Flow_{i,j}\right)$	$ln\left(Flow_{i,j}\right)$	$ln\left(Flow_{i,j}\right)$	$ln\left(Flow_{i,j}\right)$
$\Delta Job_{i,j}$	0.305 ***	1.04	0.183 ***	1.14
	(0.009)		(0.005)	
$\Delta Living_{i,j}$	0.005	1.18	0.036 ***	1.05
	(0.006)		(0.007)	
$\Delta Job_{i,j}*\Delta Living_{i,j}$	−0.003	1.01	−0.003 *	1.02
	(0.002)		(0.002)	
$Dist_{i,j}$	−0.963 ***	1.10	−0.791 ***	1.11
	(0.029)		(0.027)	
$ln\left(Pop_{i}\right)$	0.887 ***	1.12	0.943 ***	1.06
	(0.022)		(0.022)	
$Terr_{j}$	−0.493 ***	1.20	−0.657 ***	1.14
	(0.057)		(0.052)	
Constant	−7.511 ***		−8.046 ***	
	(0.369)		(0.381)	
Observations	3762		3779	
R-squared	0.595		0.583	

Robust standard errors are in parentheses. *** *p* < 0.01, ** *p* < 0.05, * *p* < 0.1.

**Table 4:** The robust check for model (2) by adding a dummy variable.

Year	2010		2020	
	(1)	(2)	(3)	(4)
	$ln\left(Flow_{i,j}\right)$	$ln\left(Flow_{i,j}\right)$	$ln\left(Flow_{i,j}\right)$	$ln\left(Flow_{i,j}\right)$
$\Delta Job_{i,j}$	0.303 ***	1.04	0.179 ***	1.02
	(0.009)		(0.005)	
$\Delta Living_{i,j}$	0.009	1.18	0.030 ***	1.06
	(0.006)		(0.007)	
$\Delta Job_{i,j}*\Delta Living_{i,j}$	−0.003	1.01	−0.003 *	1.02
	(0.002)		(0.002)	
$Dist_{i,j}$	−0.978 ***	1.10	−0.833 ***	1.14
	(0.029)		(0.027)	
$ln\left(Pop_{i}\right)$	0.882 ***	1.12	0.935 ***	1.06
	(0.022)		(0.022)	
$Terr_{j}$	−0.468 ***	1.20	−0.674 ***	1.14
	(0.057)		(0.052)	
$Disaster_{j}$	0.465 ***	1.01	0.521 ***	1.04
	(0.055)		(0.049)	
Constant	−7.503 ***		−7.965 ***	
	(0.366)		(0.376)	
Observations	3762		3779	
R-squared	0.595		0.583	

Robust standard errors are in parentheses. *** *p* < 0.01, ** *p* < 0.05, * *p* < 0.1.

**Table 5:** The robust regression results of model (2) by the QAP method.

Year	2010		2020	
	(1)	(2)	(3)	(4)
	$ln\left(Flow_{i,j}\right)$	*p*-Value	$ln\left(Flow_{i,j}\right)$	*p*-Value
$\Delta Job_{i,j}$	0.305 ***	0.005	0.183 ***	0.005
$\Delta Living_{i,j}$	0.005	0.448	0.036 *	0.100
$\Delta Job_{i,j}*\Delta Living_{i,j}$	−0.003	0.285	−0.003	0.274
$Dist_{i,j}$	−0.962 ***	0.005	−0.791 ***	0.005
$ln\left(Pop_{i}\right)$	0.892 ***	0.005	0.942 ***	0.005
$Terr_{j}$	−0.493 **	0.030	−0.657 ***	0.005
Constant	−7.604		−8.029	
Observations	3762		3779	
R-squared	0.589		0.583	

Robust standard errors are in parentheses. *** *p* < 0.01, ** *p* < 0.05, * *p* < 0.1.

## Data Availability

Not applicable.

## Appendix A

More details on *hukou*. *Hukou* is a unique regulation in mainland China working as a residence register and resource allocation system. One person has only one *hukou* at a place where he/she has access to local medical insurance, elementary education, the opportunity to buy a welfare house, etc., which are provided exclusively for local residence. Migrants can transfer their *hukou* to other places if they meet the standards and are accepted by the destination city government. The standards include how long migrants have worked and submitted social insurance (specific amount of money) to the destination city government. Therefore, many migrants choose their destinations for special resources, e.g., better education, and have worked hard for years. Thus, they can transfer their *hukou* there and let their family access good public resources, which are a substantial part of good living conditions.

## Appendix B

**Table A1:** Detailed description of data sources.

Variable	Description	Source
$Flow_{i,j}$	The migrants from the origin *i* to the destination *j*.	*China Population Census Yearbooks* (2010, 2020)
$Pop_{i}$	Population counts of province *i*.	
$Job_{i}$	GDP per capita, calculated as the GDP from yearbooks divided by the population $Pop_{i}$.	*China Health Statistics Yearbooks* (2011, 2021), *China Statistical Yearbook* (2011, 2021), *China County Statistical Yearbooks* (2011, 2021).
$Heal_{i}$	Number of medical beds per 1000 people, calculated as the medical beds from yearbooks divided by the population (unit/1000 persons).	
$Edu_{i}$	Number of students at elementary schools per 1 million people, calculated as the students from yearbooks divided by the population (unit/10^6^ persons).	
$Living_{i}$	Combination of $Heal_{i}$ and $Edu_{i}$.	$Living_{i}=0.808\cdot Heal_{i}+0.589\cdot Edu_{i}$
$\Delta Job_{i,j}$	Gap in jobs between the origin *i* and the destination *j*.	$\Delta Job_{i,j}=Job_{j}-Job_{i}$
$\Delta Heal_{i,j}$	Gap in medical beds between the origin *i* and the destination *j*.	$\Delta Heal_{i,j}=Heal_{j}-Job_{i}$
$\Delta Edu_{i,j}$	Gap in elementary school students between the origin *i* and the destination *j*.	$\Delta Edu_{i,j}=Edu_{j}-Edu_{i}$
$\Delta Living_{i,j}$	Gap in living conditions between the origin *i* and the destination *j*.	$\Delta Living_{i,j}=Living_{j}-Living_{i}$
$Dist_{i,j}\;\left(i\neq j\right)$	Inter-province migration distance measured by the length between the centroids of two provinces.	*Location-Based Service* by AMAP (https://lbs.amap.com/ (accessed on 15 August 2022))
$Dist_{i,j}\;\left(i=j\right)$	Intra-province migration distance measured by the square root of the area of the province *i*.	
$Terr_{j}$	Dummy variable: The provinces in which most areas are not suitable for settlement, including Tibet, Xinjiang, Ningxia, Qinghai, Chongqing, Yunnan, and Gansu.	*Website of Geospatial Data Cloud* (http://www.gscloud.cn (accessed on 24 October 2022))
$Disaster_{j}$	Dummy variable: $Disaster_{j}$ is 1 as there were natural disasters in the past ten years before the census in province j, otherwise 0. For example, if the province *j* suffered an earthquake or typhoon in 2000–2010, $Disaster_{j}$ is 1, otherwise 0 for the regression test of 2010.	Websites associated with disaster records (e.g., http://www.jnlncc.cn/life/3483415.html (accessed on 24 October 2022))
